# Measuring efficiency of governmental hospitals in Palestine using stochastic frontier analysis

**DOI:** 10.1186/s12962-016-0052-5

**Published:** 2016-02-03

**Authors:** Samer Hamidi

**Affiliations:** Chair of Health Studies Department, School of Health and Environmental Studies, Hamdan Bin Mohammed Smart University, P.O. Box 71400, Dubai, United Arab Emirates

**Keywords:** Stochastic frontier analysis, Cobb–Douglas, Translog, Technical efficiency

## Abstract

**Background:**

The Palestinian government has been under increasing pressure to improve provision of health services while seeking to effectively employ its scare resources. Governmental hospitals remain the leading costly units as they consume about 60 % of governmental health budget. A clearer understanding of the technical efficiency of hospitals is crucial to shape future health policy reforms. In this paper, we used stochastic frontier analysis to measure technical efficiency of governmental hospitals, the first of its kind nationally.

**Methods:**

We estimated maximum likelihood random-effects and time-invariant efficiency model developed by Battese and Coelli, 1988. Number of beds, number of doctors, number of nurses, and number of non-medical staff, were used as the input variables, and sum of number of treated inpatients and outpatients was used as output variable. Our dataset includes balanced panel data of 22 governmental hospitals over a period of 6 years. Cobb–Douglas function, translog function, and multi-output distance function were estimated using STATA 12.

**Results:**

The average technical efficiency of hospitals was approximately 55 %, and ranged from 28 to 91 %. Doctors and nurses appear to be the most important factors in hospital production, as 1 % increase in number of doctors, results in an increase in the production of the hospital of 0.33 and 0.51 %, respectively. If hospitals increase all inputs by 1 %, their production would increase by 0.74 %. Hospitals production process has a decrease return to scale.

**Conclusion:**

Despite continued investment in governmental hospitals, they remained relatively inefficient. Using the existing amount of resources, the amount of delivered outputs can be improved 45 % which provides insight into mismanagement of available resources. To address hospital inefficiency, it is important to increase the numbers of doctors and nurses. The number of non-medical staff should be reduced. Offering the option of early retirement, limit hiring, and transfer to primary health care centers are possible options. It is crucial to maintain a rich clinical skill-mix when implementing such measures. Adopting interventions to improve the quality of management in hospitals will improve efficiency. International benchmarking provides more insights on sources of hospital inefficiency.

## Background

Occupied Palestinian territories (OPT) consist of two geographically separated areas, West Bank (WB) and Gaza Strip (GS), and is administered by the National Palestinian Authority. OPT cover an area of about 6860 km^2^ (6500 km^2^ in WB and 360 km^2^ in GS). The total population of OPT in 2013 was about 4.5 million inhabitants with 50.8 % of males and 49.2 % of females. Demographic distribution indicates that the society is very young, about 41 % of inhabitants are under 15 years. OPT comprise of 16 governorates and are very densely populated country, with more than 650 inhabitants per square kilometer. Between 1980 and 2013, life expectancy at birth increased by about 10.4 years to reach 72.6 years. The crude death rate per 1000 inhabitants has decreased from 4.1 in 1993 to 2.5 in 2013. The infant mortality rate per 1000 live births was also decreased from 32 in 1993 to 19 in 2013. OPT are in transition politically as well as epidemiologically. OPT are suffering the double burden of both infectious and chronic diseases. The total number of full time health workforce in 2011 was about 23,888, of which 68 % employed in WB and 32 % in GS, where Palestinian Ministry of Health (MOH) employs about 60 % of them. The number of doctors and nurses per capita increased substantially in OPT over the past two decades, to reach 24 physicians per 10,000 inhabitants and 25 nurses per 10,000 inhabitants in 2013 [1].

Over the last two decades the Palestinian government carried out concrete steps to increase the effectiveness and efficiency and contain the cost of hospitals in OPT. MOH is the main entity responsible to govern, regulate, deliver services, and finance health system. The total bed capacity in 2013 was 5619 beds, which can be translated into 13 beds per 10,000 inhabitants. Beds are distributed in 80 hospitals; 30 are in WB with 3263 beds, making up 58 % of total hospital beds, the remainder is in GS. About 70 % of hospitals and 47 % hospital beds in OPT are private and non-for-profit. MOH owns and operates 53 % of total hospital bed capacity (2979 beds) distributed in 24 hospitals [1]. Within governmental hospitals, there are considerable differences from hospital to hospital. Some smaller hospitals providing only basic specialist care, the other hospitals are specialty hospitals, which limit their care to selective illnesses or patient groups. The average occupancy rate in MOH hospitals is estimated at 85 % in the WB and 78 % in the GS. There are disparities between regions in terms of occupancy rate in the governmental hospitals. The occupancy rate for all Palestinian hospitals, however, is estimated at 65 %; indicating that there is under-utilized service capacity in private sector.

Health spending as percentage of GDP increased from 9.2 % in 2000 to 12.3 % in 2011 [2]. This percentage is higher than any country in the region, and in fact very few countries in the world spend more than this percentage. While there is a positive correlation between spending on health and income per capita, higher spending observed in OPT does not seem primarily attributable to greater income. CHE increased from $384.3 million in 2000 to $1262 million in 2012, and CHE per capita grew from $137 in 2000 to $308 in 2011, a 226 % increase, while GDP per capita increased from $1498 to $2506 in the same years, a 167 % increase. Based on national income and number of population, a linear regression would predict that OPT health spending would be about $229 per capita or 9.1 % of GDP, far less than is actually observed. In fact, CHE had increased markedly from 2000 to 2011, driven by increasing salaries to finance excessive health sector employment, cost of pharmaceuticals, and outsourced health services [3]. About quarter of budget of MOH was spent on health services outsourced from other providers, and about half of the budget was spent on salaries. Hospitals remain the leading costly units in the Palestinian health system. On average hospitals consumed about 36 % of current health expenditures (CHE) during the period 2000–2011. However, governmental hospitals consumed about 60 % of MOH budget, yet inferior to private hospitals in terms of efficiency and quality [4]. In most OECD countries, hospitals also accounted for the highest share of CHE, on average (36 %), ranging from 26 % in Slovakia to 45 % in Denmark. Hospitals in Qatar and Dubai accounted for about 40 %, and 48 % of CHE, respectively [5].

The economic challenges in terms of high rate of poverty and limited financial support; and the political challenges presented by Israeli occupation atrocities against the Palestinian people, the separation and fragmentation of the Palestinian communities, and closures remain the main determinant of health in OPT. The ongoing conflict with Israeli occupation forces caused measurable deterioration in health status and health services delivery as a result of constrained access to health facilities, health professionals, medical equipment, and pharmaceuticals. The construction of the Apartheid Wall which encloses about 120,000 inhabitants and hinders their access to hospitals, and confiscates their livelihood of living, forcing them into poverty. The separation of health care delivery between WB and GS, and control of all movement, complicate the ability of MOH to coordinate its activities, often leading to duplication and loss of efficiency. Besides, most of hospitals are allocated inside cities and a lot of people face difficulties to reach hospitals especially in Alquds (Jerusalem). Hospitals are overwhelmed by the number of casualties, and its inability to keep up with housekeeping and sterilization has increased the rate of infections reported after discharge from hospitals [3].

The main objective of the study is to measure technical efficiency of governmental hospitals and quantify the effects of number of beds, doctors, nurses and non-medical staff on their technical efficiency.

## Literature review

It has been well established in literature that inefficiencies in health spending are large [6]. World Health Organization (WHO) estimates that about 20–40 % of resources spent on health are wasted [7]. The most common causes of inefficiency include inappropriate and ineffective use of medicines, medical errors, suboptimal quality of care, waste, corruption, and fraud [8]. Because of inefficiencies, many countries could achieve the same level of health outcomes with a lower level of spending [9]. Hospital productivity is one measure of the effective use of resources and measures outputs relative to the inputs needed to produce them. Efficiency is the degree to which the observed use of resources to produce outputs of a given quality matches the optimal use of resources to produce outputs of a given quality. So, efficiency is a component of productivity and refers to the comparison between actual and optimal amounts of inputs and products. In general, efficiency is productivity adjusted for the impact of environmental factors on performance. Effectiveness is the extent to which outputs of service providers meet the objectives set for them. Economists distinguish among three main measures of efficiency namely technical, allocative and total economic efficiency. Technical efficiency refers to the manner in which resources are employed so as to lead to the greatest level of output. As such technical efficiency emphasizes the technological aspects of an organization. In the case of hospital technical efficiency implies how the inputs which are essentially the physical assets, labor and financial resources are used to produce both intermediate and final outputs whereby examples of the former include number of patients, waiting time and so on while the latter include mortality rates, quality of life measures and so on [10]. Scale efficiency is a component of technical efficiency. Constant returns to scale (RTS) signify perfect scale efficiency. If a hospital is operating at either increasing or decreasing RTS, it is not scale efficient [11]. Allocative efficiency refers how an organization is able to use inputs in an optimal manner based on their respective prices and technology. As such allocative efficiency measures how an organization is able to select the optimal combination of inputs to produce the greatest level of outputs. Total economic efficiency which is the combined impact of technical and allocative efficiency.

The literature to date has tended to use a number of different methods to estimate the efficiency of hospitals. In some cases the measures of efficiency are influenced by government policy. A typical example of this is the UK where the National Health Service has developed efficiency performance indicators and labour productivity measures to benchmark the different providers so as to produce rankings [12]. The problem with efficiency measures is that their selection can be subjective, and the final value is highly dependent on the weights used. A more objective and economics based approach is to estimate the production possibility frontier (PPF) which is a locus of potentially efficient output combinations that an organization can employ at a particular point in time. The PPF is the most used method to estimate the efficiency of hospitals. The PPF is considered the efficient frontier as any hospital production at that level is able to achieve an efficient combination of inputs. Similarly, a hospital that does not produce on the efficient frontier is considered to be technically inefficient.

There is no consensus on the best method to measure technical efficiency. Previous studies have identified two methods, namely, non-parametric methods initiated by Charnes et al. (1978) [13] and a parametric technique developed by Aigner (1977) [14]. Parametric methods focus on economic optimization, while non-parametric techniques examine technological optimization. The most common estimation technique under the nonparametric approach is the data envelopment analysis (DEA). The major advantage of DEA is that it avoids having to measure output prices which are not available for transactions and services and fee based outputs. However, DEA method is non-stochastic and does not capture random noise such as strikes, and any deviation from the estimated frontier is interpreted as being due to inefficiency. With DEA also it is not possible to conduct statistical tests of the hypothesis regarding the inefficiencies scores.

On the other hand, the main models under parametric approach include Stochastic Frontier Analysis (SFA) of Battesse and Coelli (1992; 1995) [15, 16], and Huang and Liu (1994) [17]. While DEA does not separate out the effects of a stochastic error term, SFA disentangles the two sources of error, due to inefficiency and random noise. In SFA approaches it is possible to conduct statistical tests of the hypothesis regarding the inefficiencies scores. The main advantage of SFA is that it accounts for the traditional random error of regression. SFA presents a production function of the standard regression model but with a composite disturbance term equal to the sum of the two errors components [14, 18].

The stochastic frontier production function indicates the existence of technical inefficiency of production [16, 19]. The stochastic frontier divides the distance to the frontier into random error and inefficiency. The random error takes into account exogenous shocks. Criticisms of SFA include the need to specify in advance the mathematical form of the production function and the distributional form of the inefficiency term.

SFA is a parametric technique of frontier estimation that assumes a given functional form for the relationship between inputs and an output [20]. Some SFA modeling approaches of panel data assume a uniform variation for all Decision Making Units (DMUs) such as Battese and Coelli (1992) [16], others such as Greene (2005) [21] allow for stochastic variation without any correlation over time. The latter models include three stochastic components respectively for efficiency, random noise, and time-invariant heterogeneity. Goudarzi et al. (2014) used SFA method was applied to estimate the efficiency of twelve teaching hospitals by analyzing a 12-year panel data, and founded remarkable waste of resources [22].

Output-oriented distance function is used to measure the difference between potential and observed output, usually denoted as technical inefficiency. The distance from an observation to the frontier is the measure of technical efficiency. Gerdtham, et al. (1999) used multiple-output stochastic ray analysis and panel data on 26 hospitals over 7 years to investigate the effect of reimbursement reform on technical efficiency [23]. Ferrari (2006) used distance functions and panel data on 52 hospitals over 6 years to evaluate the impact of introducing internal competition on technical efficiency [24]. Daidone and D’Amico (2009) adopted a distance function approach, while measuring the technical efficiency level with stochastic frontier techniques. They evaluated how the productive structure and level of specialization of a hospital affect technical efficiency by analyzing a 6-year panel data [25].

## Methods

### Data

This paper analyzed technical efficiency of governmental hospitals, selected on the basis of most recently available comparable data. Our dataset includes balanced panel data of 22 governmental hospitals over a period of 6 years, 2006, 2007 and 2009–2012, providing 132 observations. The two governmental psychiatric hospitals were excluded because their inputs and outputs are different from other hospitals. Data were not available for year 2008. Data were collected from the annual reports of the MOH from 2006, 2007, and 2009–2012 [26–31]. The variables used are defined in Table 1, along with summary statistics. The data consists of inputs to hospital production in the form of capital and labour, and outputs from production. Labour inputs are measured by the number of people employed in each hospital and we use full-time equivalent staff to measure labour input. Four input variables were included in the efficiency analysis (1) the number of hospital beds in the year in each hospital was used as an index of capital input (2) the number of Full Time Equivalent (FTE) doctors (3) the number of FTE nurses (4) the number of FTE non-medical which included all staff other than nurses and doctors. The categorization of health workforce to three categories of doctors, nurses, and non-medical staff was due to the evidence that these categories of resources have different roles in patient care and deliver services [22].

**Table 1:** Descriptive statistics of the input and output variables

Variable	Hospital average value per year	Hospital median value per year	Standard deviation	Minimum	Maximum
Number of beds	120	101	106	10	509
Number of doctors	90	53	119	11	652
Number of nurses	119	95	93	2	436
Number of non-medical staff	152	115	115	25	537
Total staff	361	282	311	47	1561
Number of admitted patients	14,045	9348	12,171	294	55,519
Average length of stay (ALOS)	2.4	2.4	0.7	1.0	5.1
Number of hospital days	33,896	23,180	31,465	895	150,486
Number of operations	3357	1785	4445	0	26,516
Number of outpatient visits	99,765	78,444	103,578	11,169	539,914

Source (MOH 2006, 2007, 2009, 2010, 2011, 2012)

The outputs of hospital production consist of the sum of inpatients and outpatients in each hospital. The emergency visits were considered also as outpatient visits. The inpatients were measured as the total number of admitted patients within a year. Outpatients are counted as total yearly number of attendances at outpatient clinics in each hospital. Standard SFA models are limited to only one output. This limitation necessitates aggregation of inpatient and outpatient workload into one variable. Since the 22 hospitals are very different in terms of size and kind of provided health care, the sum of number of treated inpatients and outpatients might not be adequate. For example, one hospital can have a lower efficiency score because of the mix of products in terms of specialization and not because of resource misuse. To address this issue we used multi-output distance function model within the SFA to estimate technical efficiency.

### Modelling

Two forms of production function most used is literature to measure hospital inefficiency are the Cobb–Douglas and translog functional forms. In previous studies of hospital efficiency the parametric production function has been represented by a Cobb–Douglas function [32], representing unitary elasticity of substitution. While the Cobb–Douglas form is easy to estimate, its main drawback is that it assumes constant input elasticities and RTS for all hospitals. On the other hand, the translog form does not impose these restrictions but is susceptible to degrees of freedom problems and multicollinearity. In this study we estimated three models: Cobb–Douglas form, translog form, multi-output distance form. The three models used the normal-truncated normal maximum likelihood (ML) random model effect with time-invariant efficiency developed by Battese and Coelli 1988 [33]. The models were estimated by using xtfrontier command of STATA 12.

The empirical model of Cobb–Douglas function form is given by Eq. 1.

1 $$ln\left( {y_{it} } \right) = \beta_{0} + \sum_{j = 1}^{k} {\beta_{j }\, ln \,x_{j,it} + \left( {V_{it } - U_{it} } \right)}$$

where j is the number of independent variables, i is the decision making units (hospitals), t is the time in years. Ln represents the natural logarithm, y_it_ represents the output of the i-th hospital at time t, *x*_*jit*_ is the corresponding level of input j of the i-th hospital at time t, β is a vector of unknown parameters to be estimated. The *v*_*it*_ is a symmetric random error, to account for statistical noise with zero mean and unknown variance σv2. The *u*_*it*_ is the non-negative random variable associated with technical inefficiency of hospital i, its mean is mi and its variance is σu2.

We tailored the Cobb–Douglas function form to the purpose of the current study. So, the Cobb–Douglas production function form is presented in Eq. 2.

2 $$ln\left( {Outpatient_{it} + Inpatient_{it} } \right) = \beta_{0} + \beta_{1 } \,lnBed_{it} + \beta_{2 }\,lnDoctor_{it} + \beta_{3 }\,lnNurse_{it} + \beta_{4 }\,lnNonmedical_{it}$$

The translog function is very commonly used and it is a generalization of the Cobb–Douglas function. It is a flexible functional form providing a second order approximation. The empirical model of translog function form is given by Eq. 3.

3 $$ln\left( {y_{it} } \right) = \beta_{0} + \sum_{j = 1}^{k} {\beta_{j }\,ln x_{j,it} } + \frac{1}{2}\sum_{j = 1}^{k} {\sum_{h = 1}^{k} {\beta_{jh } \,lnx_{jit}\,lnx_{hit} + \left( {V_{it } - U_{it} } \right)} }$$

where j is the number of independent variables, i is the decision making units (hospitals), t is the time in years. ln represents the natural logarithm, y_it_ represents the output of the i-th hospital at time t, x_jit_ is the corresponding level of input j of the i-th hospital at time t, x_jit_ times x_hit_ is the interaction of the corresponding level of inputs j and h of the i-th hospital at time t, β is a vector of unknown parameters to be estimated. The *v*_*it*_ is a symmetric random error, to account for statistical noise with zero mean and unknown variance σv2. The *u*_*it*_ is the non-negative random variable associated with technical inefficiency of hospital i, its mean is mi and its variance is σu2.

We tailored the translog function form to the purpose of the current study as presented in Eq. 4.

4 $$\begin{aligned} ln\left( {Outpatient_{it} + Inpatient_{it} } \right) = \beta_{0 } + \beta_{1 }\,lnBed_{it} + \beta_{2 }\,lnDoctor_{it} + \beta_{3 }\,lnNurse_{it} \hfill \\ \;\;\;\;\;\;\;\;\;\;\;\;\;\;\;\;\;\;\;\;\;\;\;\;\;\;\;\;\;\;\;\;\;\;\;\;\;\;\;\;\;\;\;\; + \beta_{4 }\,lnNonmedical_{it} + \beta_{12 }\, \left( {lnBed_{it} \times\,lnDoctor_{it} } \right) + \beta_{13 }\, \left( {lnBed_{it} \times lnNurse_{it} } \right) \hfill \\ \;\;\;\;\;\;\;\;\;\;\;\;\;\;\;\;\;\;\;\;\;\;\;\;\;\;\;\;\;\;\;\;\;\;\;\;\;\;\;\;\;\;\;\; + \beta_{14 }\, \left( {lnBed_{it} \times lnNonmedical_{it} } \right) + \beta_{23 }\, \left( {lnDoctor_{it} \times lnNurse_{it} } \right) \hfill \\ \;\;\;\;\;\;\;\;\;\;\;\;\;\;\;\;\;\;\;\;\;\;\;\;\;\;\;\;\;\;\;\;\;\;\;\;\;\;\;\;\;\;\;\; + \beta_{24 }\, \left( {lnDoctor_{it} \times lnNonmedical_{it} } \right) + \beta_{34 }\, \left( {lnNurse_{it} \times lnNonmedical_{it} } \right) \hfill \\ \;\;\;\;\;\;\;\;\;\;\;\;\;\;\;\;\;\;\;\;\;\;\;\;\;\;\;\;\;\;\;\;\;\;\;\;\;\;\;\;\;\;\;\; + \beta_{11 } \,0.5\,\left( {lnBed_{it} \times lnBed_{it} } \right) + \beta_{22 }\, 0.5\left( {lnDoctor_{it} \times lnDoctor_{it} } \right) \hfill \\ \;\;\;\;\;\;\;\;\;\;\;\;\;\;\;\;\;\;\;\;\;\;\;\;\;\;\;\;\;\;\;\;\;\;\;\;\;\;\;\;\;\;\;\; + \beta_{33 } \, 0.5\,\left( {lnNurse_{it} \times lnNurse_{it} } \right) + \beta_{44 }\, 0.5\,\left( {lnNonmedical_{it} \times lnNonmedical_{it} } \right) \hfill \\ \end{aligned}$$

where, β0 is the intercept of the constant term, β1, β2, β3, β4 are first order derivatives, β11, β22, β33, β44 are own second order derivatives and β12, β13, β14, β23, β24, β34, are cross second order derivatives. As a double log form model (where both the dependent and explanatory variables are in natural logs), the estimated coefficients show elasticities between dependent and explanatory variables. The stochastic frontier production function and the technical inefficiency models are jointly estimated by the maximum-likelihood method. We tested the null hypothesis that the Cobb–Douglas function form is an adequate representation of the data.

When using a translog production function the values of the input coefficients themselves do not have an easily interpretable meaning, so to truly assess input effects the marginal effects for each input were estimated using Eq. 5, where the marginal product is equal to the elasticity of scale for each input.

5 $$e_{j } = \frac{{\partial ln\left( {y_{i} } \right)}}{{\partial ln\left( {x_{ji} } \right)}} = \beta_{j } + \sum_{j = 1}^{4} {\beta_{jh} \ln x_{j} + \beta_{jt} }$$

The third model estimated was the multi-output distance function. Using a multi-output distance function will allow the specified model of hospital production and inefficiency to be explored without aggregating inpatient and outpatient visits. We tailored the multi-output distance function to the purpose of the current study as presented in Eq. 6.

6 $$\begin{aligned} ln\left( {Outpatient_{it} } \right) = \beta_{0 } + \beta_{1 }\,lnBed_{it} + \beta_{2 }\,lnDoctor_{it} + \beta_{3 }\,lnNurs + \beta_{4 }\,lnNonmedical_{it} \hfill \\ \;\;\;\;\;\;\;\;\;\;\;\;\;\;\;\;\;\;\;\;\;\;\;\;\;\;\; + \beta_{12 }\, \left( {lnBed_{it} \times lnDoctor_{it} } \right) + \beta_{13 }\, \left( {lnBed_{it} \times\,lnNurse_{it} } \right) \hfill \\ \;\;\;\;\;\;\;\;\;\;\;\;\;\;\;\;\;\;\;\;\;\;\;\;\;\;\; + \beta_{14 }\, \left( {lnBed_{it} \times lnNonmedical_{it} } \right) + \beta_{23 }\, \left( {lnDoctor_{it} \times lnNurse_{it} } \right) \hfill \\ \;\;\;\;\;\;\;\;\;\;\;\;\;\;\;\;\;\;\;\;\;\;\;\;\;\;\; + \beta_{24 }\, \left( {lnDoctor_{it} \times lnNonmedical_{it} } \right) + \beta_{34 }\, \left( {lnNurse_{it} \times lnNonmedical_{it} } \right) \hfill \\ \;\;\;\;\;\;\;\;\;\;\;\;\;\;\;\;\;\;\;\;\;\;\;\;\;\;\; + \beta_{11 } \,0.5\,\left( {lnBed_{it} \times lnBed_{it} } \right) + \beta_{22 } \,0.5\,\left( {lnDoctor_{it} \times lnDoctor_{it} } \right) \hfill \\ \;\;\;\;\;\;\;\;\;\;\;\;\;\;\;\;\;\;\;\;\;\;\;\;\;\;\; + \beta_{33 } \,0.5\,\left( {lnNurse_{it} \times lnNurse_{it} } \right) + \beta_{44 } \,0.5\,\left( {lnNonmedical_{it} \times lnNonmedical_{it} } \right) \hfill \\ \;\;\;\;\;\;\;\;\;\;\;\;\;\;\;\;\;\;\;\;\;\;\;\;\;\;\; + \beta_{5 } { \ln }Y_{it}^{*} + \beta_{51 }\, { \ln }\left( {lnBed_{it} \times { \ln }Y_{it}^{*} } \right) + \beta_{52 }\, { \ln }\left( {lnDoctor_{it} \times { \ln }Y_{it}^{*} } \right) \hfill \\ \;\;\;\;\;\;\;\;\;\;\;\;\;\;\;\;\;\;\;\;\;\;\;\;\;\;\; + \beta_{53 } \,{ \ln }\left( {lnNurse_{it} \times { \ln }Y_{it}^{*} } \right) + \beta_{54 }\, { \ln }\left( {lnnonmedical_{it} \times { \ln }Y_{it}^{*} } \right) + \beta_{55 } \,0.5\,{ \ln }\left( {{ \ln }Y_{it}^{*} \times { \ln }Y_{it}^{*} } \right) \hfill \\ \end{aligned}$$

where, Y* is the ratio of outpatient visits to inpatient admissions. β5, is first order derivative, β55 are own second order derivatives and β51, β52, β53, β54, are cross second order derivatives.

#### Estimating technical efficiency

The technical efficiency of a hospital is defined as a ratio of the observed output (Y_*it*_) to the maximum feasible output, defined by a certain level of inputs used by the hospital. Thus, the technical efficiency of hospital *i* at time *t* can be expressed in Eq. 7.

7 $$TE_{it } = E \left[ {\exp {{\left( { - u_{it} } \right)} \mathord{\left/ {\vphantom {{\left( { - u_{it} } \right)} {\left( {v_{it} - u_{it} } \right)}}} \right. \kern-0pt} {\left( {v_{it} - u_{it} } \right)}}} \right]$$

U_*it*_ represents hospital specific fixed effects or time invariant technical inefficiency and *V*_*it*_ is a normally distributed random error term and is uncorrelated with the explanatory (independent) variables.

Since *U*_*it*_ is a nonnegative random variable, these technical inefficiencies lie between 0 and unity, where unity indicates that this firm is technically efficient. The value of *U*_*it*_ is positive and it decreases the efficiency of an object, therefore we have −*U*_*it*_. The method of ML is used for estimation of the unknown parameters, with the stochastic frontier and the inefficiency effects estimated simultaneously. Maximum feasible output is determined by the firms with inefficiency effect equal to 0 (*v*_*it*_ = 0). Equation 7 was estimated by E {exp (−s**u** [it] |**e** [it]) following Battese and Coelli, 1988, using the Te option of STATA 12.

## Results and discussion

### Descriptive analysis

Before interpreting the results of SFA and technical efficiency, the descriptive analysis of input variables and output is presented in Table 1. The annual average number of inpatient admissions per hospital was about 14,045. The annual average number of outpatient visits per hospital was about 99,765. There were on annual basis per hospital approximately, 120 beds, 90 doctors, 119 nurses, and 152 non-medical staff.

**Fig. 1:**
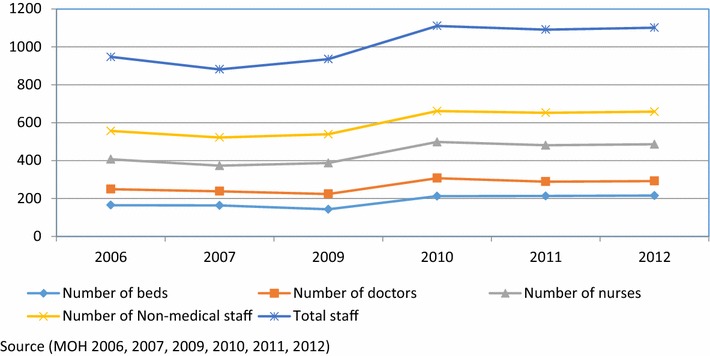
Number of beds, doctors, nurses and non-medical staff per hospital 2006–2012

The average number of all inputs remained almost unchanged from 2006 till 2009. There is a noticed increase in all inputs in 2009, then no increase is noticed in 2010–2012, as shown in Fig. 1.

### Hypothesis testing

1. The first step regarding the suitable stochastic frontier model tests revolved on the validity of the translog over the Cobb–Douglas specification within the ML specifications using null hypothesis H0: β11 = β22 = β33 = β12 = β13 = β14 = β23 = β24 = β34 = 0. The degrees of freedom of 10 and critical value of 18.3, so the null hypothesis was rejected, and it was concluded that translog form (LR = 28.886, p < 0.0001) was more appropriate for the stochastic frontier model compared to Cobb–Douglas form (LR = 9.975, p < 0.0001).
2. The second step is testing if there is significant technical inefficiency using null hypothesis H0: γ = 0, which tests whether the observed variations in efficiency are simply random or systematic. If gamma ϒ is close to zero, the differences in the production will be entirely related to statistical noise, while if gamma ϒ close to one reveals the presence of technical inefficiency. The estimate of parameter ϒ (0.792), which measures the variability of the two sources of error, suggests 79 % of the total variation of total production related to inefficient error term and 21 % of the total variation attributed to the stochastic random errors. This implies that the variation of the total production among the different hospitals was due to the differences in their production inefficiencies, indicating that traditional production function ordinary least squares (OLS) is not an adequate representation of our data. We applied the log-likelihood ratio test to assess whether SFA should be used instead of OLS. The null hypothesis that the OLS regression was as appropriate as SFA was rejected indicating that inefficiency effects should be included. The presence of inefficiency is also confirmed by the high values of the contribution of the inefficiency (u) to the total error.
3. The third step of testing concerned the distribution of the inefficiency effects using null hypothesis H0: μ = 0. The null hypothesis specifies that each hospital is operating on the technical efficient frontier and that the asymmetric and random technical efficiency in the inefficiency effects are zero. The null hypothesis that the technical inefficiency effects have a half-normal distribution (i.e., μ = 0), was rejected against the null that the technical inefficiency effects have a truncated normal distribution. The coefficient of mu (μ = 0.627; p < 0.05) is the estimate of μ, the mean of the truncated normal distribution (the mean of the error component relative to inefficiency) is statistically signif-icant, indicating that the normal truncated distribution is more appropriate than the half-normal distribution. These results are also confirmed by the comparing values of the variances of technical inefficiency term (sigma_u2) and variance of random error (sigma_v2).

### Stochastic frontier analysis

The ML estimates of stochastic frontier production function were obtained applying the normal-truncated normal ML random-effects model with time-invariant efficiency developed by Battese and Coelli 1988 [33]. The results obtained with the Cobb–Douglas and translog, and multi-output distance functions are presented in Table 2. The focus was on a stochastic frontier model which assumes time invariant inefficiencies. This was done because the length of the panel is short and we hoped not to confound the time trend capturing productivity change with that capturing efficiency change. The first section of Table 2 presents frontier function with four parameters for Cobb–Douglas function, fourteen parameters for translog function, and 20 parameters for multi-output distance function. The second section presents the variance parameters, the amount of the function of the log likelihood, and the likelihood ratio (LR) test. Table 2 reports estimates of parameters sigma_u2 (0.086), sigma_v2 (0.022), sigma2 (0.109), lnsigma2 (−2.216), gamma (0.792), ilgtgamma (1.338), and mu (0.627). Sigma_u2 (0.086) is the estimate of δu2. Sigma_v2 (0.022) is the estimate of δv2. Sigma2 (0.109) is the estimate of δs2 = δu2 + δv2. Because δs2 must be positive, the optimization is parameterized in terms of ln (δs2), and this estimate is reported as lnsigma2 (−2.216). Gamma is the estimate of ϒ = δu2/δs2. Because ϒ must be between 0 and 1, the optimization is parameterized in terms of the inverse logit of ϒ, and this estimate is reported as ilgtgamma (1.338).

**Table 2:** Maximum likelihood estimates of the stochastic frontier models

Ln (output)	Parameter	Cobb–Douglas function	Translog function	Multi-output distance function
Constant	β0	8.601***	12.446***	11.49***
Ln (bed)	β1	0.621***	1.607*	−1.07
Ln (doctor)	β2	0.263**	2.351*	3.47**
Ln (nurse)	β3	−0.039	−0.360	0.31
Ln (non-medical staff)	β4	−0.031	−3.945*	−2.46
Ln (bed) × ln (doctor)	β12		−0.915**	−1.27***
Ln (bed) × ln (nurse)	β13		0.023	−0.51
Ln (bed) × ln (non-medical staff)	β14		0.452	1.58***
Ln (doctor) × ln (nurse)	β23		1.306***	1.58***
Ln (doctor) × ln (non-medical staff)	β24		−1.890***	−1.97***
Ln (nurse) × ln (non-medical staff)	β34		−1.203**	−1.01**
Ln (bed) × ln (bed)	β11		0.00004	0.27
Ln (doctor) × ln (doctor)	β22		0.663***	1.40***
Ln (nurse) × ln (nurse)	β33		0.137*	0.26
Ln (non-medical staff) × ln (non-medical staff)	β44		1.526***	1.19
Ln (outpatient/inpatient)	β5			−1.37
Ln (outpatient/inpatient) × ln (bed)	β51			−0.10*
Ln (outpatient/inpatient) × ln (doctor)	β52			0.07
Ln (outpatient/inpatient) × ln (nurse)	β53			−0.47
Ln (outpatient/inpatient) × ln (non-medical staff)	β54			−0.18
Ln (outpatient/inpatient) × ln (outpatient/inpatient)	β55			0.98**
Variance of technical inefficiency (sigma_u2)	δu2	0.128	0.086	0.284
Variance of random error (sigma_v2)	δv2	0.030	0.022	0.145
Sigma square (sigma2)	δs2 = δu2 + δv2	0.158	0.109	0.429
Ln sigma square (lnsigma2)	Ln (δs2)	−1.840***	−2.216***	−2.27***
Variance ratio parameter (gamma)	ϒ = δu2/δs2	0.807	0.792	0.662
Inverse logit gamma (ilgtgamma) = 0	ilgt ϒ	1.435*	1.338**	1.34**
mu	μ	0.563*	0.627**	0.671**
Wald Chi square (4, 14)	χ2	138.158***	285.563***	379.66***
Number of observations	N	132	132	132

* p < 0.05; ** p < 0.01; *** p < 0.001

#### Output elasticities of input variable

We concluded earlier that translog model is more appropriate to present our data. The results of translog model indicates that the first order coefficients are not conclusive as they do not provide much information on the responsiveness of the output to the various inputs. Based on this argument, output elasticities of each of the inputs at their mean values were calculated using Eq. 5 as shown in Table 3.

**Table 3:** Output elasticities of input variables (Scale elasticity)

Inputs	Scale elasticity
Number of beds	0.15
Number of doctors	0.33
Number of nurses	0.51
Number of nonmedical staff	−0.25
Total	0.74

### Technical efficiency

**Fig. 2:**
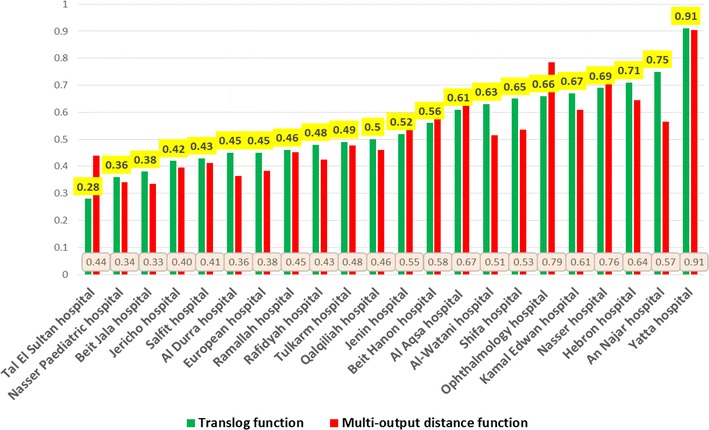
Average technical efficiencies of 22 hospitals using both translog, and multi-output distance functions

Following Battese and Coelli (1988), technical efficiencies were estimated using a one-step maximum likelihood estimates (MLE) procedure, by incorporating the model for technical efficiency effects into the production function. Because one hospital can have a lower efficiency score because of the mix of products in terms of specialization and not because of resource misuse, we estimated technical efficiency using both the translog function, and multi-distance function, and compared them, as shown in Fig. 2. Panel-data analysis allowed the enlargement of a small cross-section of 22 hospitals into a 132 observations sample over a period of 6 years.

Results from translog function revealed that the average technical efficiency of hospitals was 55 %, and ranged from 28 to 91 %, with a median of 51 %. Results from the multi-output distance function revealed that the average technical efficiency of hospitals using the multi-output distance function was 53 %, and ranged from 44 to 91 %, which indicated 47 % potential for improvement. There were no full efficient hospital during the entire study period. About 5 % of the hospitals (1 hospital) had a technical efficiency between 0.80 and 1.0, and about 36 % of the hospitals (8 hospitals) had technical efficiency between 0.6 and 0.80. About 59 % of the hospitals (13 hospitals) had technical efficiency between 0.2 and 0.6.

A paired t test was run on a sample of 22 hospitals over 6 years to determine whether there was a statistically significant mean difference between the technical efficiency when we used translog function compared to a multi-output distance function. Technical efficiency of hospitals was lower when using multi-output distance function (0.527 ± 0.15) as opposed to the translog function (0.547 ± 0.15); a statistically not significant decrease of 0.02 (95 % CI, −0.055–0.014), t = 1.2, p = 0.23. Table 4 shows the average technical efficiency scores for both standard and multi-output distance functions.

**Table 4:** Average technical efficiency scores of all 22 hospitals over period of study

Function	Average technical efficiency	Standard deviation	95 % confidence interval	
Translog function	0.55	0.15	0.52	0.57
Multi-output distance function	0.53	0.15	0.45	0.59

The study used balanced cross-sectional time-series panel data of 22 governmental hospitals over a period of 6 years. Results from Cobb–Douglas model indicates that 1 % increase in number of beds and number of doctors will results in 0.621 and 0.263 % increase in production of hospitals measured by number of treated inpatients and outpatients, respectively. Number of nurses and number of non-medical staff were not significant to production. However, we concluded from the SFA that translog model is more appropriate to present data than Cobb–Douglas model, so we will depend on the results of translog model. The first order coefficients in translog model are not conclusive as they do not provide much information on the responsiveness of the output to the various inputs, because the translog functional form used precludes normalization of the outputs in the production function to the mean vector. If the variables in the translog model were mean-corrected to zero, then the first order coefficients are the estimates of the elasticities at the mean input levels, however, they were not. Consequently, the first order coefficients on the input variables in the translog model are used to calculate the output elasticity with respect to each input in the production function at their mean values. By using mean-scaled variables, it is possible to interpret the first-order coefficients of the translog function as the partial elasticities of production for the sample mean.

The output elasticities measures the responsiveness of output to a change in inputs. Table 3 indicates the estimated output elasticities at the mean values of the inputs or scale elasticities of inputs. The measure of RTS represent the percentage change in output due to a proportional change in use of all inputs, and it is estimated as the sum of output elasticities for all inputs. If this estimate is greater than, equal to, or less than one, we have increasing, constant, or decreasing RTS respectively. These estimates were 0.15, 0.33, 0.51 and 0.25 for beds, doctors, nurses and non-medical staff, respectively. Doctors and nurses appear to be the most important factors in hospital production. Apparently doctors have positive influence on the productivity of other inputs, so that their net contribution is positive. This is consistent with the conventional notion that doctors direct the use of non-doctor resources in hospitals. Consistent with our a priori expectation, that except non-medical staff, all other three inputs make significant contributions to the optimum production scales. If there is 1 % increase in number of beds, number of doctors and number of nurses holding number of non-medical staff without change, then hospitals will have constant RTS (1.0). The sum of these elasticity coefficients is equal to 0.74, which indicates that the production process has a decrease return to scale (DRS). So, if hospitals increased all inputs by 1 %, production would increase by about 0.74 %. In other words, hospitals have not worked in the optimum production scales, and that the majority of hospitals do not fully achieve the potential scale economies.

The second order coefficients and interaction terms coefficients in translog model are almost completely statistically significant except interaction between beds and nurses, and interaction between beds and non-medical staff. Most interaction coefficients turned out to be highly significant indicating that the usage levels of the four inputs were interdependent on each other. The results of the SFA analysis shows that the number of doctors has a significant effect on the production both partially or in the form of quadratic and interaction. The consistency of the strong influence of the production through a translog SFA.

Doubling the use of inputs means using these inputs once again in the hospital for the purpose of increasing productivity. Therefore, squaring (doubling) the number of doctors, nurses, and non-medical staff increases hospital output by 0.663, 0.137, and 1.526 units per unit of output, respectively, through marginal. So, investment in doctors, nurses, and non-medical staff yields increasing return to scale. However, we noticed that the coefficient of number of non-medical staff is significant and negative while the coefficient of the square of number of non-medical staff is significant and positive. This indicates that hospitals with lower number of non-medical staff are more productive that hospitals with higher number of non-medical staff. A decrease in the number of non-medical staff in each hospital will result in the improvement of production. It is interesting to note that that the coefficient of number of doctors and the coefficient of the square of number of doctors is significant and positive. This indicates that hospitals with lower number of doctors are less productive that hospitals with higher number of doctors. An increase in the number of doctors in each hospital will result in improvement of production. We also noticed that the coefficient of number of nurses is not significant and negative and while coefficient of the square of number of nurses is significant and positive. This indicates that an increase or doubling the number of nurses will result in large improvement of hospital production.

The coefficient of interaction between beds and doctors is negative when both first order coefficients are positive. The number of beds has two effects on hospital output, through the direct effect, the number of beds directly and positively affects output, and through the indirect effect, the number of beds changes the effect of number of doctors, nurses, and non-medical staff on the output. The negative sign on interaction between beds and doctors indicates some substitutability of doctors and hospital beds. The results indicate that 1 % increase in number of beds should reduce the number of doctors required by 0.9 %. This means that in the presence of more than required beds, doctors productivity could be reduced leading to lower output level. This may reflect a higher tendency for doctors to keep patients hospitalized longer and to utilize more ancillary services, which may reduce the number of treated patients. However, the interactions of beds with nurses and non-medical staff were not significant, suggesting that inclusion of outpatients as a component in the output reduces the importance of hospital beds in the production of the hospital.

Doctors and nurses and complementary as indicated by the positive sign observed for the interaction between doctors and nurses. The first order coefficients are positive for doctors and negative for nurses. The number of doctors has two effects on hospital output. Through the direct effect, the number of doctors directly and positively affects output, and through the indirect effect, the number of doctors changes the effect of number of nurses on output.

The results indicate that 1 % increase in number of doctors should increase the number of nurses required by 1.3 %.

The number of non-medical staff has two effects on hospital output. Through the direct effect, the number of non-medical staff directly and negatively affects output, and through the indirect effect, the number of non-medical staff changes the effect of number of doctors and nurses staff on output. The negative sign on interaction between doctors and non-medical staff indicates that they are substitutes, suggesting that health care delivery in the hospital involves many other tasks than just the direct interaction of doctors and patients, and reflects the importance of non-medical staff. The results indicate that 1 % increase in number of non-medical staff should reduce the number of doctors and nurses required by 1.89 and 1.2 % respectively. Key strategies for increasing non-medical staff productivity include better management of overtime and sickness absence, and making a rich clinical skill-mix when reducing the overall numbers.

The average technical efficiency of hospitals calculated by standard translog function was 55 %, and ranged from 28 to 91 %, which indicated 45 % potential for improvement through more effective use of the input bundle given the present state of technology. These technical efficiency scores are comparable to those revealed by Goudarzi et al. (2014) [22] where technical efficiency was about 59 %. However, the average technical efficiency score is considerably low compared with efficiency of hospitals in Saudi Arabia with technical efficiency score of 84.6 % [34], and OECD counties with technical efficiency scores range from 62 to 96 % [35]. A study in Netherlands demonstrates that the average efficiency for Dutch hospitals is 84 % [36].

## Limitations of the study

Similar to previous studies on hospital efficiency, our study suffers several limitations. First, because a simple empirical model was used in this study, there is a possibility of the omitted variables problem, which may bias the estimation of time-invariant component of hospital production efficiency. Also the inputs and outputs used in this research allowed us to perform efficiency analyses of hospitals, but we should also recognize their weaknesses. The number of beds is used to proxy capital inputs, but hospitals may also use different technologies, so we assumed that the comparison hospitals use similar levels of technology. Also, using the number of beds instead of active beds may result in huge differences across hospitals in efficiency in terms of occupancy rates and duration of stay. On the other hand, the labour input we use is a good standard measure and sufficiently captures the variation of labour inputs between hospitals. Turning to the output measures, the output measure is not adjusted for quality or case mix, and differences in the severity of cases may affect the number of cases hospitals dealt with relative to their staff numbers and could therefore have an impact on the results of the analysis. Research highlights the need for using case mix adjusted data in analyzing hospital efficiency [37]. Second, a relatively small sample size and a short time interval of 6 years may limit the generalizability and estimation efficiency of our results. Despite our best efforts to obtain the necessary information to construct our production function model, data in panel were only available for 6 years and for governmental hospitals. Data were not available for private hospitals. As a result, our sample is not representative of all OPT hospitals. Finally, the model outlined above following Battese and Coelli, 1988 [28] assumes that the inefficiency effects are time invariant. In a panel with many years, this assumption may be questionable, so we wish to return to this topic in future research to estimate a model that assumes time-varying inefficiency for comparison purposes. Despite this, there is evidence to suggest that there are considerable efficiency gains yes to be made by MOH.

## Conclusions

Technical efficiency analysis is used as a review tool to assess decisions regarding allocation of human and capital resources. This study measured technical efficiency of governmental hospitals using SFA. The average technical efficiency of governmental hospital was approximately 55 %, and about 45 % the production factors are wasted during the service delivery process in the hospitals. Using the existing amount of resources, the amount of delivered outputs can be doubled, which can significantly impact patient outcomes. Despite continued government investment in the hospital sector through capital hospital expansion, hiring workforce, and promotion of new technology, hospitals has remained relatively inefficient. The efficiency scores provide insight into mismanagement of available resources. Improving efficiency while containing cost, is a key policy challenge in OPT. Higher spending on hospitals will not necessarily translate into effective results if spending is not directed towards the most cost-effective interventions. A variety of strategic options are available, and governmental hospitals show varying capacity to adopt these options. To address inefficiency in hospitals, policy makers may increase output in terms of treated patients, reduce inputs, and change organization and processes in hospitals. Interventions to improve the quality of management in hospitals could help to improve efficiency. International benchmarking of hospital efficiency help to provide more insights on sources of hospital inefficiency. Given the positive effect of increasing number of number of doctors and nurses on efficiency. However, key strategies for increasing non-medical staff efficiency include reducing their numbers. Offering the option of early retirement, limit hiring, and transfer to primary health care centers are possible options. It is crucial to maintain a rich clinical skill-mix teams of health workforce, and effectively manage overtime and sickness absence when implementing such measures.

This article was an attempt to measure technical efficiency of governmental hospitals in OPT to inform future health policy making and health planning. Internationally, the results contribute to the growing literature on SFA methodology. It is also an invitation to other researchers in the field to apply other quantitative techniques to provide deep insight into how governmental and private hospitals manage their human and capital resources. Only this kind of understanding could help us to be sure that we are moving forward in our journey to enhance the efficiency of the health system.
